# Supplementary material for: WordCommentsAnalyzer: A windows software tool for qualitative research
Source: F1000Res. 2018 Sep 4;7:536. Originally published 2018 May 3. [Version 2] doi: 10.12688/f1000research.14819.2 (PMC6137411; doi:10.12688/f1000research.14819.2)
Supplement: Supplementary file 3 [file f1000research-7-17614-s0002.tgz › b2a79496-4ada-47c4-9682-a99ce867836d.zip!/Eight Iranian Health Journals/Salmand_Iranian Journal of Ageing _23.docx]

© Salmand_Iranian Journal of Ageing . This work is licensed under CC BY-NC 4.0 (https://creativecommons.org/licenses/by-nc/4.0/).

Volume & Issue: دوره 7 - شماره 3

Title: بررسی ارتباط واریاسیون‌های ژن‌های APOE و TNF-α با بیماری آلزایمر اسپورادیک در جمعیت ایرانی

http://salmandj.uswr.ac.ir/article-1-338-fa.html

اهداف: مطالعات متعدد مشخص کرده علاوه بر رسوب دو پروتئین پپتید بتا آمیلوئید و neurofibrillary tangles که مکانیسم های اصلی در پاتوژنز بیماری آلزایمر محسوب می‌شوند، مکانیسم های التهابی نیز درپاتوژنز این بیماری نقش اساسی دارند. واسطه‌های التهابی متنوع چون فعال کننده‌ها و مهارکننده‌های کمپلمان، کموکاین‌ها و سیتوکین‌ها که توسط میکروگلیاها و آستروسیت‌ها رها می شوند، سبب نقص در عملکرد و مرگ تدریجی نورونها می‌گردند. یکی از مهم‌ترین این سیتوکین‌ها، فاکتور تومور نکروز آلفا TNF-α می باشد. این مطالعه به منظور بررسی رابطه بین واریانت‌های ژن‌های TNF-α و APOE با بیماری آلزایمر اسپورادیک در جمعیت ایرانی، طراحی و اجرا گردید. مواد و روش‌ها: در این مطالعه مورد– شاهدی، پلی مورفیسم ژن TNF-α در 163 فرد سالم و 167 فرد مبتلا به آلزایمر نوع اسپورادیک مورد بررسی قرار گرفت. DNA ژنومی با روش Salting Out از لنفوسیت‌های خون محیطی استخراج شده و ژنوتایپینگ نمونه‌ها برای پلی مورفیسم C>T در پروموتر ژن TNF-α با استفاده از روش PCR/RFLP و الکتروفورز پلی آکریل آمید انجام شد. نتایج به دست آمده با استفاده از تستهای مجذور کای و رگرسیون لجیستیک با کمک نرم‌افزار SPSS 11/5 مورد تجزیه و تحلیل آماری قرار گرفتند. یافته‌ها: نتایج به دست آمده نشان دهنده فراوانی بیشتر معنی‌دار ژنوتیپ TNF-α -850 C/T در جمعیت مورد بیمار نسبت به گروه کنترل بوده است (P=0/038). گرچه تفاوت معنی داری در فراوانی ژنوتیپ هوموزیگوت TNF-α -850 T/T و نیز در فراوانی آللی TNF-α -850 Tمشاهده نگردید (P>0/05). در اثرمتقابل دو پلی مورفیسم ژن‌های TNF-α و APOE نیز نتیجه معنی داری بدست نیامد (P>0/05). نتیجه‌گیری: این مطالعه پلی مورفیسم هتروزیگوت C/T TNF-α -850 را بعنوان ریسک فاکتور برای بیماری آلزایمر اسپورادیک در جمعیت ایرانی نشان می‌دهد. برای نشان دادن اثر پلی مورفیسم هوموزیگوتT/T TNF-α -850 و آلل T، مطالعات آتی با حجم نمونه بیشتر پیشنهاد می‌شود.

Title: خواب‌آلودگی روزانه: عاملی مرتبط با سلامت روان سالمندان؟

http://salmandj.uswr.ac.ir/article-1-517-fa.html

اهداف: در دهه‌های اخیر، به‌دلیل افزایش نرخ رشد جمعیت سالمندی در دنیا، مسائل بهداشتی این گروه نیز اهمیت بیشتری یافته است. ازآنجا‌که بهداشت روان و اختلالات خواب از مشکلات مهم دوران سالمندی است، این مطالعه با هدف بررسی ارتباط سلامت روان و خواب‌آلودگی روزانۀ سالمندانی انجام گرفت که به سازمان بازنشستگی کشور مراجعه‌ می‌کنند. مواد و روش‌ها: در این تحقیق توصیفی‌تحلیلی در تهران، ۳۹۰ سالمند مراجعه‌کننده به سازمان بازنشستگی و ادارات وابسته‌به آن، در سال ۱۳۸۶ به روش نمونه‌گیری آسان دردسترس و با استفاده از سه پرسش‌نامۀ اطلاعات جمعیت‌شناختی و خواب‌آلودگی (Epworth) و سلامت عمومی (GHQ) پانزده‌سؤالی بررسی شدند. اطلاعات جمع‌آوری‌شده، با استفاده از روش‌های آماری توصیفی تی تست، کای دو، و آنوا تجزیه‌و‌تحلیل شد. یافته‌ها: ازمیان آزمودنی‌ها، ۵۱درصد مرد، اکثریت آنان متأهل (۶۶/۸درصد) و بیشتر آنان در ردۀ سنی ۶۴-۶۰ سال بودند (۴۹درصد). همچنین ۲۹ درصد کل آزمودنی‌ها خواب‌آلودگی خفیف تا شدید داشتند و ۴۶درصد آنان مشکوک به مشکلات سلامت روان تشخیص داده شدند. سلامت روان به‌عنوان فاکتوری مستقل، با خواب‌آلودگی سالمندان ارتباط آماری معنی‌داری داشت (۰/۰۵>P). نتیجه‌گیری: با توجه به ارتباط معنی‌دار سلامت روان و خواب‌آلودگی در سالمندان، توصیه می‌شود برای بررسی دقیق‌تر این ارتباط و تعیین تقدم و تأخر هرکدام از این عوامل نسبت‌به یکدیگر، مطالعات تکمیلی صورت گیرد.

Title: تأثیر خاطره‌گویی گروهی بر معیار ناتوانی در سالمندی، در سالمندان ساکن در خانه‌های سالمندان شهر مشهد، در سال ۱۳۹۰

http://salmandj.uswr.ac.ir/article-1-503-fa.html

اهداف: هدف از تحقیق حاضر، تعیین تأثیر خاطره‌گویی گروهی بر معیار ناتوانیِ سالمندان ساکن در خانه‌های سالمندان شهر مشهد بود. مواد و روش‌ها: در این مطالعۀ نیمه‌تجربی، ۵۶ نفر از سالمندان ساکن در خانه‌های سالمندان شهر مشهد به روش در دسترس انتخاب شدند. واحدهای پژوهشگر هریک از خانه‌های سالمندان، به‌صورت غیرتصادفی به گروه خاطره‌گویی گروهی (۳۰ نفر) و کنترل (۲۶ نفر) اختصاص داده شدند. به‌این‌ترتیب، ۶ گروه خاطره‌گویی ۴تا۸ نفری تشکیل شد. خاطره‌گویی گروهی، در مدت ۸ هفته، هفتگی برگزار شد و هر جلسه حدود یک ساعت طول کشید. معیار ناتوانی در سالمندی، افسردگی، عملکرد‌شناختی و درد، به‌ترتیب با استفاده از نسخۀ دوم پرسش‌نامۀ سنجش ناتوانی سازمان بهداشت جهانی، مقیاس ۱۵ آیتمی افسردگی سالمندان، آزمون خلاصه‌شدۀ وضعیت روانی و مقیاس دیداری درد اندازه‌گیری شدند. برای تجزیه و تحلیل داده‌ها، از نرم‌افزار SPSS، نسخۀ ۵/۱۱ و آمار توصیفی و استنباطی استفاده شد. یافته‌ها: نتیجۀ آزمون‌های آنالیز واریانس با مقادیر تکراری و من‌ویتنی، نشان داد بین تغییرات معیار ناتوانی در سالمندی (P=0/94) و زیرمجموعه‌های آن؛ یعنی برقراری ارتباط و درک‌مطلب (P=0/20) تحرک و جابه‌جایی (P=0/20)، انجام فعالیت‌های خودمراقبتی (P=0/92) سازش و زندگی‌کردن با دیگران (P=0/72) و مشارکت (P=0/88) در دو گروه کنترل و خاطره‌گویی گروهی تفاوت آماری معناداری وجود نداشت. نتیجه‌گیری: برگزاری ۸ جلسۀ هفتگیِ یک ساعتی برای خاطره‌گویی گروهی، معیار ناتوانی در سالمندی و زیرمجموعه‌های آن را در سالمندان ساکن در خانه‌های سالمندان کاهش نداد. بنابراین، مطالعات بیشتر در این زمینه توصیه می‌شود.

Title: اثر 3 ماه تمرین مقاومتی بر سطوح سرمی پروتئین واکنشی-C و هیپرتروفی عضلانی در مردان سالمند

http://salmandj.uswr.ac.ir/article-1-408-fa.html

اهداف: سارکوپنیا کاهش توده و قدرت عضلانی مرتبط با افزایش سن است. شواهد پیشنهاد می‌کند که التهاب نقش مهمی درآتروفی عضلانی مرتبط با افزایش سن بازی می‌کند. هدف مطالعه حاضر بررسی اثر 3 ماه تمرین مقاومتی بر توده عضله اسکلتی و سطوح پروتئین واکنشی-C در مردان سالمند بود. مواد و روش‌ها: در این مطالعه نیمه تجربی با طرح پیش آزمون – پس آزمون، بیست و پنج مرد سالمند (سن: 3/40±64/10 سال، نمایه توده بدنی: 2/38±28/29 کیلوگرم بر متر مربع) بطور تصادفی به دو گروه تمرین مقاومتی (13 نفر) و کنترل (12 نفر) اختصاص داده شدند. برنامه تمرین مقاومتی 60-50 دقیقه در روز، سه روز در هفته و برای 3 ماه اجرا شد. سطوح سرمی پروتئین واکنشی-C و ترکیب بدنی (DEXA) قبل و بعد از مداخله اندازه‌گیری گردید. یافته‌ها: بعد از تمرین مقاومتی، پرس پا (شاخص قدرت پایین تنه)، پرس سینه (شاخص قدرت بالا تنه) و توده عضله اسکلتی به‌طور معنی‌دار افزایش یافت (0/05>P)، درحالیکه شاخص‌های چربی بدن (ازجمله نمایه توده بدن و توده چربی) در پاسخ به تمرین مقاومتی تغییر نکرد (P>0/05). همزمان، سطوح پروتئین واکنشی-C در گروه تمرین بطور معنی‌دار کاهش یافت (0/05>P). نتیجه‌گیری: سه ماه تمرین مقاومتی موجب بهبود قدرت و توده عضلانی در مردان سالمند می‌شود و این بهبودی با کاهش در سطوح سرمی پروتئین واکنشی-C همراه بود.

Title: تقارن در پارامترهای منتخب توزیع فشار کف پایی سالمندان دارای کوتاهی پا (LLD)

http://salmandj.uswr.ac.ir/article-1-519-fa.html

اهداف: کوتاهی اندام تحتانی (LLD)، از مشکلاتی است که تغییراتی در پارامترهای مختلف ازجمله توزیع فشار کف پایی حین گام‌برداری به وجود می‌آورد. هدف این تحقیق، بررسی تقارن در پارامترهای منتخب توزیع فشار کف پایی سالمندان دارای کوتاهی پا (LLD) بود. مواد و روش‌ها: در این تحقیق، ۲۱ سالمند دارای کوتاهی اندام (1.5تا3 سانتی‌متر) از شهرستان اصفهان به‌صورت نمونه مشارکت کردند. فشار کف پایی و پارامترهای مرتبط با آن با استفاده از پلت‌فرم emed، مدل emed 2، در ۵ گام اندازه‌گیری شد. از گام‌های یادشده، برای هر پا میانگین‌سه گام گرفته شد. پس از تقسیم کف پا به ۱۱ ناحیه، حداکثر فشار، نیرو، سطح تماس، زمان تماس، انتگرال فشار-زمان و انتگرال نیرو-زمان در هر ناحیه محاسبه شد. از آمار توصیفی (میانگین و انحراف استاندارد) برای گزارش رکوردها، از آزمون تی وابسته برای مقایسۀ پارامترهای پای کوتاه و بلند (P<0/05) و از شاخص تقارن (%SI) برای بررسی وضعیت تقارن در پارامترهای منتخب فشار کف پایی استفاده شد. یافته‌ها: نتایج آزمون تی وابسته، ‌به‌جز سطح تماس در ناحیۀ جلوی پا و انگشتان ۳و۴و۵ (0/05>P) هیچ‌گونه اختلاف معناداری را بین دو سمت کوتاه و بلند نشان نداد. شاخص تقارن (%SI) نیز نشان داد که در ناحیۀ پاشنۀ سمت کوتاه زمان تماس کمتر، در ناحیۀ وسط پای سمت کوتاه، نیرو و فشار کمتر و در ناحیۀ جلوی پای کوتاه، فشار بیشتر بوده است. نتیجه‌گیری: براساس نتایج این تحقیق، زمان تماس اولیه و پذیرش وزن در پای کوتاه کاهش می‌یابد. این فرایند، موجب افزایش فشار ناحیۀ جلوی پا در مرحلۀ قبل از پوش‌آف شده و متاتارسال‌ها را در معرض خطر استرس فرکچر قرار می‌دهد. برای حل این مشکل و اصلاح الگوی گام‌برداری پای کوتاه، استفاده از کفی‌هایی پیشنهاد می‌شود که ارتفاع معقولانه‌ای به پاشنه داده، درنتیجه زمان تماس می‌دهند.

Title: نقش جنسیت بر میزان پروتئین مایواستاتین پلاسما در حا‌لت استراحت و درپا‌سخ به فعالیت حاد مقاومتی در مردان و زنان سالمند

http://salmandj.uswr.ac.ir/article-1-537-fa.html

اهداف: هدف از این مطالعه بررسی نقش جنسیت بر میزان پروتئین مایواستاتین پلاسما در حالت استراحت ودرپاسخ به فعالیت حاد مقاومتی در مردان وزنان سالمند بود. مواد و روش‌ها: در این مطالعه 12 مرد (سن 1/23±69 سال و شاخص توده بدنی kg/m2  23±0/3) و 12 زن (سن 2/14±68 سال و شاخص توده بدنی kg/m2 23±0/4) سالمند سالم، به صورت داوطلبا‌نه شرکت داشتند. 72 ساعت پس از تعیین حداکثر قدرت بیشینه (با استفاده از آزمون 1-RM)، آزمودنی ها دریک جلسه مقاومتی با شدت 75درصد 1-RM شرکت نمودند. در این پژوهش 3 نمونه خونی در قبل، بلافاصله و 4 ساعت بعد ازفعالیت مقاومتی از ورید بازویی آزمودنی ها گرفته شد. به منظور تعیین میزان مایواستاتین پلاسما از روش الایزا استفاده شد. جهت تجزیه وتحلیل آماری داده های تحقیق از نرم افزار SPSS نسخه 16 استفاده شد. سطح معناداری P≤0/05 در نظر گرفته شد. یافته‌ها: نتایج تحقیق حاضر نشان داد که میزان مایواستاتین پلاسما در حالت استراحت در زنان به طور معناداری بالاتر از مردان است(t(22)= -10/90, P=0/001). همچنین میزان مایواستاتین پلاسما در بلافاصله و چهار ساعت بعد از فعالیت، در هردوگروه مردان وزنان، کاهش معناداری یافت (P≤0/05). در هردوگروه میزان مایواستاتین پلاسما در چهار ساعت بعد از فعالیت نسبت به بلافاصله بعد از فعالیت کاهش یافت، اما این کاهش معنادار نبود. بین گروه ها نیز از لحاظ پاسخ مایواستاتین پلاسما به فعالیت مقاومتی، تفاوت معناداری مشاهده نشد. نتیجه‌گیری: یافته‌های این پژوهش نشان داد که میزان پروتئین مایواستاتین پلاسما در حالت استراحت در زنان سالمند به طور معناداری بالاتر از مردان سالمند می‌باشد. اما میزان این پروتئین در هردوگروه در پاسخ به فعالیت مقاومتی (دربلافاصله و چهارساعت بعد از فعالیت) کاهش معناداری یافت. همچنین نشان داده شد که جنسیت بر پاسخ مایواستاتین پلاسما به فعالیت مقاومتی درمردان وزنان سالمند، عامل اثرگذاری نمی باشد. بنابریافته های این تحقیق به نظر می رسد که با اعمال یک محرک تمرینی مشابه، می‌توان موجب کاهش مشابه مایواستاتین و متعاقباً فرآیند سارکوپنیا در هردوگروه مردان و زنان سالمند شد.

Title: بررسی تأثیر به‌کارگیری روش توسعه مددکاری گروهی با تمرکز بر آموزش مهارت‌های زندگی بر بهبود کیفیت زندگی و سلامت عمومی سالمندان فرهنگسرای سالمند تهران

http://salmandj.uswr.ac.ir/article-1-198-fa.html

اهداف: هدف این تحقیق، شناخت تأثیر مداخله مددکاری اجتماعی با استفاده از مدل توسعه مددکاری گروهـی در قالب آموزش مهارت‌های زندگی بر بهبود کیفیت زندگی و سلامت عمومی سالمندان بود.  مواد و روش‌ها: در این پژوهشِ مداخله‌ای و شبه‌آزمایـشی، 60 سـالمند بـیش از 60 سـال (30 زن و 30 مـرد) از فرهنگسرای سالمند تهران با احتساب معیارهای ورود و خروج و با استفاده از روش نمونه‌گیری در دسترس، در دو گروه مرد و زن برگزیده شدند. مداخله‌ای مبتنی بر مدل توسـعه مـددکاری گروهـی در دو مرحلـه و طـی 10 جلسه اجرا شد که عبارت‌اند از: 1. تشکیل گروه؛ 2. اجرای برنامه آموزشی مهارت‌هـای زنـدگی. اطلاعـات بـا استفاده از پرس‌شنامه‌های دموگرافیک و کیفیت زندگی 38-sf و سلامت عمومی 28-GHQ با روش پیش‌آزمـون و پس‌آزمون به‌دست آمد. برای تحلیل داده‌ها از spss 18 و آزمون‌های آماری کلمـوگرف-اسـمیرنف، تـی مـستقل و وابسته استفاده شد.  یافته‌ها: کیفیت زندگی دو گروه جنسی شرکت‌کننده در مطالعه با یکدیگر متفاوت بـود و کیفیـت زنـدگی زنـان به‌طور معناداری بیشتر بود (0/002=P). میانگین کیفیت زندگی سالمندان بعد از شرکت در دوره آموزشی افـزایش معناداری یافت (0/00=P). از نظر سلامت عمومی، تفاوت معناداری میان دو گروه جنسی دیده نـشد (0/99=P) و میانگین سلامت عمومی سالمندان نیز پس از شرکت در دوره آموزشی از شرکت در دوره افزایش معناداری یافت (P=0/00). نتیجه‌گیری: مطالعه حاضر نشان داد به‌کارگیری دیدگاه توسعه مددکاری گروهی با رویکرد آموزش مهارت‌های زندگی، بر کیفیت زندگی و سلامت عمومی دو گروه جنسی سالمندان تأثیر مثبت داشته است. لذا، استفاده از ایـن مدل برای مددکاران اجتماعی شاغل، به‌ویژه مددکاران سالمندی، توصیه می‌شود.

Title: وضعیت اضطراب و افسردگی و احساس خوب‌بودن روانی، در سالمندان شهری منطقۀ تحت پوشش دانشگاه علوم پزشکی شهیدبهشتی در تهران

http://salmandj.uswr.ac.ir/article-1-445-fa.html

اهداف: این پژوهش به بررسی مؤلفه‌های سلامت روان، ازقبیل اضطراب و افسردگی و احساس روانی خوب‌بودن، در گروه‌های مختلف سنی سالمندان ساکن شهر تهران می‌پردازد. دستاوردهای این مطالعه، با تعیین میزان اختلالات روانی، در تبیین اولویت‌های مراقبتی در گروه‌های سنی سالمندان، به سیاست‌گذاری‌های بهداشتی ضروری آنان کمک می‌کند. مواد و روش‌ها: این مطالعۀ توصیفی‌تحلیلی، به روش تصادفی، در سال ۱۳۸۹، دربارۀ ۴۰۲ سالمند شصت‌ساله و بیشترِ ساکن شهر تهران، در منطقۀ تحت پوشش دانشگاه علوم‌پزشکی شهید بهشتی انجام گرفت. پرسش‌نامه‌ها به روش مصاحبۀ چهره‌به‌چهره و در چند نمونۀ خاص، با مصاحبۀ تلفنی تکمیل شد. ابزارهای جمع‌آوری داده‌های پرسش‌نامه، در سه قسمت اطلاعات دموگرافیک و تست ابزار سنجش اختلالات اضطراب و افسردگی (Kessler-K6) و پرسش‌نامۀ تست احساس خوب‌بودن روانی Yeild بوده است. برای تجزیه‌و‌تحلیل داده‌ها از نرم‌افزار SPSS نسخۀ ۱۵ و نیز نرم‌افزار Stata و آزمون‌های آماری تست تی، one-way ANOVA و مدل رگرسیون رتبه‌ای چند متغیره (ordered regression polytomos) استفاده شده است. یافته‌ها: نتایج نشان داد که در چهار هفتۀ گذشته ۱۰/۹درصد سالمندان، همیشه یا اکثر اوقات اختلالات اضطراب و افسردگی شدیدی داشته و حدود نیمی از سالمندان ۴۶٫۵درصد، ازنظر اختلالات اضطراب و افسردگی، در حد متوسطی قرار داشتند. از ۶۰ تا ۷۹سالگی، با افزایش سن سالمند، میزان اختلالات اضطراب و افسردگی نیز افزایش یافت؛ درحالی‌که این میزان، از سن ۸۰ سال به بالا روند نزولی داشت. به‌جز گروه سنی اول (۶۹-۶۰ سال) در سایر سنین، میزان اختلالات اضطراب و افسردگی زنان بیشتر از مردان بود. در بررسی احساس خوب‌بودن روانی، به‌ترتیب، احساس کسالت و اظهار تنهایی و احساس افسردگی، از شکایات بسیار شایع‌تر سالمندان بود. کمتر از نیمی از سالمندان (۴۳/۱درصد) ازنظر احساس روانی خوب‌بودن، در حد متوسط بوده و حدود ۱۷درصد در حد بد و نامناسب بودند. نتایج آنالیز رگرسیون نشان داد که متغیر‌های جنس (۰/۰۱۲=P) و وضعیت مسکن (P=۰/۰۰۴) و بازنشستگی همراه با مستمری (P=۰/۰۴۸) به‌طور معنی‌داری به‌مثابه متغیرهای تأثیرگذار بر اختلالات اضطراب و افسردگی سالمندان تحت مطالعه بودند. فقط متغیر میزان درآمد سالمندان، بر میزان احساس خوب‌بودن روانی تأثیر معنی‌داری داشت. درمقایسۀ شاخص‌های اختلالات اضطراب و افسردگی و احساس خوب‌بودن روانی، تفاوت معنی‌داری بین سالمندان دو گروه سنی ۶۴-۶۰ و ۶۹-۶۵ ساله به‌دست نیامد. تمام اختلالات یا بیماری‌های مزمن تحت بررسی بر میزان اختلالات اضطراب و افسردگی سالمندان تأثیرگذار بودند؛ درحالی‌که فقط بیماری‌های عضلانی و اسکلتی و درد و خستگی، با احساس خوب‌بودن روانی ارتباط معنی‌داری داشتند. نتیجه‌گیری: عوامل مهم‌ مؤثر بر مشکلات روحی و روانی سالمندان، با میزان درآمد آنان ارتباط دارد. به‌عبارتی زنان و سالمندانِ بدون مسکن که هنوز در خانه‌های استیجاری سکونت دارند، سالمندانی که درآمد خود را کافی نمی‌دانند، افراد کم سواد و بدون درآمد و سالمندانی که تنها زندگی می‌کنند، استرس و مشکلات روحی و اضطراب و افسردگی بیشتری دارند و احساس خوب‌بودن روانی در آنان کمتر است.
